# Psychodrama’s Role in Alleviating Acute Distress: A Case Study of an Open Therapy Group in a Psychiatric Inpatient Ward

**DOI:** 10.3389/fpsyg.2018.02075

**Published:** 2018-10-30

**Authors:** Yiftach Ron

**Affiliations:** ^1^School of Creative Arts Therapies, Kibbutzim College, Tel Aviv, Israel; ^2^Hebrew University of Jerusalem, Jerusalem, Israel

**Keywords:** psychiatric hospitalization, group therapy, acute distress, inpatients, mental illness, case study, psychodrama

## Abstract

Numerous studies point to the acute distress associated with experiencing severe mental illness and psychiatric hospitalization. Another strand of research describes how the unique features of psychodrama group therapy are useful in fostering spontaneity and creativity, and their benefits in treating particularly difficult populations where traditional psychotherapy is limited. This paper provides a framework for understanding the potential of psychodrama group therapy to alleviate the experience of loneliness and distress in psychiatric inpatients. A case study of an open inpatients psychodrama group in a psychiatric hospital in Israel demonstrates the role of therapeutic means such as the doubling technique and group sharing phase in creating and reinforcing empathy, relatedness, and support, which may offer at least partial relief of the distress and loneliness of psychiatric inpatients. The unique contribution of this study is the intimate encounter that it provides to researchers and practitioners with the processes that take place within the setting of inpatients therapy group.

## Introduction

The case study presented in this paper is intended to serve as a framework for understanding the potential of psychodrama group therapy to alleviate the experience of loneliness and distress in psychiatric inpatients. In contrast to many other studies on mental illness treatment, this study does not discuss the phenomenological aspects of psychopathology or etiological explanations based on psychoanalytical theories; rather, it focuses on the experience of participants coping with mental illness and psychiatric hospitalization. This approach follows a wider trend of psychodrama practitioners (Kellermann, 1998) and specialists in the field of psychiatric rehabilitation who prefer a more holistic, experience-based approach as an alternative to the psychopatologizing and labeling language (Roe and Lachman, 2005; Mankiewicz et al., 2013).

### Coping With Mental Illness and Psychiatric Hospitalization

The experience of coping with mental illness has been described, among others, by Patricia Deegan, one of the forerunners of the *recovery movement*. Deegan describes how a diagnosis of mental illness “paints” a person’s entire perception and subjective experience. She describes the social isolation, the feeling of failure, the detachment and alienation, which takes over a person following his or her diagnosis (Deegan, 2004). Other studies illustrate how awareness of the disease or medical diagnosis, in itself elicits symptoms of depression (Moore et al., 1999; Roe and Lachman, 2005) or even of post-traumatic stress in people coping with severe mental illness (SMI; Frame and Morrison, 2001; Mueser et al., 2002; Shaw et al., 2002). All this is intensified by the effects of stigmatization and social rejection, and in particular by self-stigmatization, further exacerbating the difficulty of coping with mental illness (Corrigan, 2004; Mueser et al., 2010; Corrigan and Rao, 2012). Self-stigma results in a loss of identity, it shapes one’s attitudes to recovery, provokes a sense of hopelessness and low self-esteem, and leads to social withdrawal and depletion of social connections (Yanos et al., 2011; Orkibi et al., 2014).

Moreover, the hospitalization and treatment experience is often in itself a traumatic experience. Recovering people undergo difficult treatments, involuntary hospitalization, sometimes they must be physically restrained or placed in isolation. They are medicated, sometimes involuntarily, suffering adverse effects which can result in a feeling of alienation from one’s own recognizable self, and may be also subject to verbal threats as well as physical, sexual, or emotional abuse by other inpatients or staff members (Morrison et al., 2003; Holloway, 2010).

It is in this challenging context that various therapy groups, such as psychodrama group therapy, are offered to people hospitalized in a psychiatric hospital.

### Group Therapy in Psychiatric Wards

Many studies have revealed the immense benefit of group therapy in people coping with shared distress, especially people with SMI (Kanas, 1996; Whitaker, 2001; Shulman, 2006; Fagin, 2010). Coping with SMI is often accompanied by a loss of internal and interpersonal dialog, and a personal experience of one-dimensionality and emptiness. In such situations, individuals feel empty and hollow, see themselves as outside observers of their own lives. People suffering from these conditions can benefit from therapy that conjures diverse situations of rich dialog, offering partners to mirror one another, and providing visibility and a voice to convey the individual’s inner narrative (Lysaker and Lysaker, 2004).

Existing literature deals with the conditions and limitations which affect the nature of group therapy for psychiatric inpatients. These include the physical limitations of a psychiatric ward, the participants’ state of acute distress, incidence of violence and self-injury (Holloway, 2010), and a high turnover of group members. This constant turnover dictates the group’s *open nature*, with participants joining and leaving frequently throughout the course of the group (Turner, 2011), and its *open-ended nature* with regard to the duration of the group (Schopler and Galinsky, 1984/2006; Miller and Mason, 2012)^1^, which Ebenstein and others categorize with *single-session groups* (Ebenstein, 1998; Manor, 2010). Yalom (1983, 1995) details the circumstances surrounding group therapy in psychiatric wards. He describes power-struggles that arise between the various members of medical and para-medical professions, the rapidly changing group membership due to increasingly shorter hospitalizations, and the psychopathological heterogeneity in the various departments. Other factors include the multiplicity of groups and activities offered to inpatients in the ward, the time spent together when outside the group, and the norms for sharing information among staff-members.

Despite these limitations, Yalom describes the benefits that group therapy may offer to psychiatric inpatients. In addition to Yalom’s definition of 11 “therapeutic factors” of group therapy in general (such as instilling hope, universality, altruism, etc.), he highlights a few goals for group therapy specifically within an inpatient ward. These include encouraging people to be involved in their treatment and recovery process, encouraging a desire to maintain and continue with treatment even after discharge; demonstrating to people that talking about one’s problems and sharing can help; identifying problems with the help of the group, in order to work on them later in one-on-one therapy; alleviating loneliness and bridging barriers between fellow inpatients; providing a tool for inpatients to help and support one other, which empowers the participants, giving them a sense of capability and self-value; and lastly, easing the anxiety that is experienced in psychiatric hospitals by offering a safe and protected space within it (Yalom, 1983, 1995).

### Psychodrama Therapy for People With Mental Illness

The psychodramatic stage allows individuals to approach their feelings and thoughts in situations where the verbal dialog of analytic psychotherapy is limited (Farmer, 1995). Psychodrama group therapy is especially beneficial for evoking spontaneity and uncovering creativity in emotional distress cases (Holmes and Karp, 1991; Roine, 1997; Blatner, 2000; Schacht, 2007; McVea et al., 2011) and in treating difficult populations, such as at-risk adolescents, alcoholics, drug addicts, sexual offenders, and those coping with anorexia (Karatas, 2011; Karp, 1994; Hollander and Craig, 2013; Orkibi et al., 2017). Another advantage of psychodrama is its effectiveness in treating a wide range of psychopathologies, including depression, anxiety disorders, obsessive compulsive disorder, post-traumatic stress disorder, and other related phenomena such as self-stigma in people coping with mental illness (Vieira and Risques, 2007; Belil, 2010; Gatta et al., 2010; Orkibi et al., 2014).

J. L. Moreno, the originator of Psychodrama, applied it with people who have SMI. He notes that Freud has consistently stated that psychoanalytic therapy is applicable only to individuals capable of evoking transference toward the analyst, and thus precludes the possibility of psychoanalytic treatment for people with psychosis and those with severe narcissistic disorders. Moreno, however, argues that such disorders do not preclude the possibility of treatment by using psychodramatic therapy (Fox, 2008). Moreno’s conception of working with people with SMI is detailed in an article dealing with the treatment of psychosis through psychodrama (Moreno, 1939 in Fox, 2008). The focus of the psychodramatic therapeutic process is not, according to Moreno, a transference of the patient to the therapist, but rather the encounter between people and the psychodramatic roles, which underlies the value of this method for people with SMI and psychotic states. Psychodrama makes use of the “tele,” the emotion that arises in interpersonal encounters and the interactions of different roles, in order to induce the recovery process even in people with severe illness unaddressed by Freudian psychoanalysis.

Through the reality on the stage, psychodramatic techniques allow a person with psychosis to express even delusions and hallucinations in an accepting way that legitimizes this experience by allowing for expression of the world as he or she experiences it. These techniques do not reinforce people’s delusions or undermine reality, but rather allow them to self-restrain the psychosis (Moreno, 1939).

Moreno describes techniques for psychodrama specifically designed to be accessible even for people with SMI that may not be capable of participate in the regular therapeutic process (Moreno, 1939; Ron, 2018). For example, the *substituting role technique* provides participants to take on symbolic roles very distant from their own selves if they are unable to play the role of themselves or those close to them. Similarly, the *mirror technique* enables even those participants who have trouble acting out a role on the psychodrama stage, to “see themselves” on stage using another group member who acts as an “auxiliary ego” in their place. For those who cannot participate whatsoever in psychodrama, Moreno suggests another technique: the *auxiliary world* technique, wherein a person’s entire daily environment becomes the psychodrama stage, and all the people in it are included by means of becoming “auxiliary egos” (actors in the main character’s drama).

This auxiliary world technique is intended primarily for psychiatric inpatients in extreme and uncommon conditions (Moreno, 1939). In practice, psychodrama therapy is utilized routinely in many psychiatric wards in Israel. This study presents findings from psychodrama group therapy in an acute ward of a psychiatric hospital in Israel and examines how group psychodrama may, to some extent, alleviate the distress and loneliness experienced by psychiatric inpatients in acute states.

## Materials and Methods

### Case Study Research

The origins of contemporary case study research can be found in qualitative approaches to research in the disciplines of anthropology, history, psychology, and sociology (Harrison et al., 2017). The term “case study” has been widely used across multiple disciplines and has no single definition (Kazdin, 2011). Yin (2009, p. 18) defines case study as “an empirical inquiry that investigates a contemporary phenomenon within its real-life context.”. Crowe and her colleagues similarly describe the case study approach as a research approach that is used to generate an in-depth multifaceted understanding of complex issues in their real-life context (Crowe et al., 2011). What is common to these various definitions is the consistent description of case study research as a versatile form of qualitative inquiry most suitable for a holistic and in-depth investigation of complex phenomena (Harrison et al., 2017). Explanatory in nature, the case study research is expected to catch the uniqueness and complexity of a single case in a real life setting (Stake, 1995). Its naturalistic and uncontrolled characteristics have made the case study a unique source of information that contributes to theory, research and practice (Kazdin, 2011; Harrison et al., 2017).

### The Study Setting and Participants

This study was conducted using the case study approach, following a group psychodrama therapy led by the author over a period of a year in the acute ward of a psychiatric hospital in Israel. People admitted to this ward are adults over age 20 suffering from some acute crisis leading them to be hospitalized voluntarily for a period lasting usually from 1 to 3 months. The circumstances leading to their hospitalization were varied, including depression, disorders such as schizophrenia and bipolar disorder, anxiety and other stress disorders, as well as various psychotic states.

The psychodrama group was an open group, allowing for high turnover and variability of the group’s participants (Turner, 2011; Miller and Mason, 2012). The study followed this group in 2011–2012 for nearly a year, during which the group met 40 times, with 85 different participants overall. Of those, 51 were men and 34 were women. Ages ranged from 22 to over 70. The number of participants in each session ranged from 4 to 11.

The sessions were led by the author, and took place once a week in the morning, lasting about an hour. Each session began with introductions and a “group pulse-check” in which the participants shared with the group how they were. This was followed by an active warm-up, then enactment of a psychodramatic vignette, and group sharing as a closure.

### Materials and Data Analysis

Data were collected through the therapist’s (i.e., the author) direct participant observation. Materials include detailed verbatim transcripts of all the therapy sessions during the course of the study, as well as other documents including drawings and letters written by the participants during the group sessions and collected by the therapist. The verbatim transcripts were written immediately following each session by the group therapist and processed in weekly supervision sessions. All materials were used with the express consent of the group participants and with the approval of the psychiatric hospital. Materials were edited with pseudonyms and any identifying information was removed.

In line with the grounded theory approach, several stages of analysis were undertaken (Strauss and Corbin, 1998; Berg, 2004). The first phase included a thematic analysis of each transcript. The units of analysis were paragraphs or segments of text from the transcript. At the same time the entire text was also treated as a single segment. The intention was to enable the necessary dismantling of each session into specific units of content while retaining the ability to see them in their original context (Berg, 2004).

The initial analysis revealed numerous thematic categories emerging from each transcript. After rereading a given transcript, the number of categories was reduced by combining similar categories and focusing on those that emerged most relevant. Next, the transcripts were integrated based on the categories that they had in common. These categories were scrutinized again for centrality (repeated appearances across interviews) for the connections between them, and for their relevance to theory, to the study’s question (Berg, 2004; Roth, 2005).

Importantly, to cross-validate the findings all sessions transcripts were reviewed by a peer psychodrama practitioner against the findings. The analysis process revealed major thematic categories which are presented in the following section.

## Findings

The findings of the study are presented in three main thematic categories: (a) encountering and coping with manifestations of distress in the group, (b) the doubling technique in the therapeutic process, and (c) the role of group sharing. The first section is an illustration of psychiatric inpatients’ acute distress and the manner in which they expressed this distress, while the latter two sections focus on the role of specific therapeutic means in coping with this distress. The decision to focus on the doubling technique and group sharing is related to the significant role they played in creating and reinforcing empathy, relatedness, and support in the group. The quoted passages are excerpts from the verbatim transcripts written by the author immediately following the group sessions. Pseudonyms are used.

### Encountering and Coping With Manifestations of Distress in the Group

Alongside the turnover of group members throughout the year, the consistent element was the manifestation of participant’s distress – the depression, despair and fatigue, helplessness, guilt, fear, and isolation.

Almost weekly, especially during the “group pulse-check” segment at the beginning of every session, group members recounted and described a sense of depression: descriptions of sinking into despair, the apathy and dysfunction that led to their hospitalization, fears and anxiety that accompany the depression at night, sleep that is completely dependent on sleeping pills, and mornings where the depression dominates their body and mind. Mornings were described as a struggle to get up from bed, move the legs and begin the day. Young mothers related being unable to care for the children or even feel any joy from them, because of the enveloping depression. Those who were beginning to recover feared sinking once again into that depression.

It was apparent that the group activity was meaningful for the participants. This at times entailed discussion and sharing, and at times was accompanied by psychodramatic techniques such as *role-reversal* or *doubling*:

Daniel was hospitalized 2 days ago because of his depression. This has already happened to him multiple times before. Last night he didn’t sleep at all, despite his sleeping pill.

Elena also talks about her depression. She says that mornings are the hardest for her. She cries all morning, and improves throughout the day.

We discuss depression a little; about how Daniel and others have been in this situation before, and recovered from it. Daniel says, “This doesn’t mean anything about the future.”

I “double” for Daniel: “When I am depressed, I cannot see how things will get better. I can’t see the light at the end of the tunnel. In my mind I know that [in the past] it has always been temporary and I [always] got better, but when I am inside the depression, it’s hard to feel that things could be good again.”

Here, Daniel shared his story of coping with depression. This was followed by a similar story by Elena. It was an opportunity for both of them to see themselves in each other’s story. I facilitated the brief group discussion and used the doubling technique in an attempt to expand Daniel’s viewpoint, a sort of narrow “tunnel vision” that often characterizes people experiencing depression. The double was also used to find a balance between identification and confrontation: allowing Daniel to sense that his emotions and helplessness are perceived and understood, while at the same time offering an alternative point of view, by echoing memories of better times he had in the past.

The expressions of depression were often accompanied by despair, weariness, and hopelessness. These may arise either as a result of the psychiatric condition and repeated failed social interactions outside the hospital, or secondary to the hospitalization itself. A concrete example is found in one session in which participants were asked to write a letter to someone who was significant to them. One of the group members, Adam, chose to write a letter to himself:

“Letter to Adam: Adam has no more strength, I’ve lost all hope, these horrible feelings won’t let me be, and every day in this ward is hell for me. I feel I might go insane, I want to go home. Every day my mother fights with me, that I’ll agree to stay here longer. In the ward, everything feels sad and gloomy, and I just cross my fingers and hope that I’ll have the patience to last until I can be discharged.”

This expression of distress, the ability to share it with the group while they do the same, is in itself beneficial. Adam’s letter prompted an opportunity to let him experience a new perspective using role-playing, and then to simply echo Adam’s words and feelings using a double:

I [the therapist] place a chair across from Adam, and ask him to sit on it. I ask him how he would respond to himself. He says, “You need to be patient.” As he returns to his own seat I ask him if he was convinced – he says he was not. I offer a double for him – “It’s difficult, I don’t have the patience.”

Alongside the depression, despair, and fatigue, some participants also expressed suicidal thoughts and a desire to die. These were not usually concrete ideas, but their presence was apparent in the group:

Anna: “I’ve been here for 2 months already, and it’s not getting better. Every day I just want to die. But I promised myself that as long as I’m here I won’t do anything.”

Later, Anna takes one of the hoops placed in the center of the circle, and tosses it outside the circle, “banishing” it.

Anna: “Those are thoughts of suicide. I want to get rid of them. I don’t want **to want** to die.”

I, as a double to Anna: “I want to live?”

Anna hesitates.

I try an alternative double: “I want **to want** to live?”

Anna: “Yes — I want to want to live” she confirms.

Other emotions that commonly arose in the group included guilt, loneliness, and a feeling of defectiveness surrounding the “illness.” The “illness” is in quotes – not because it is not real, but rather because often the world of people coping with SMI is completely colored by shades of the illness, and is accompanied by self-labeling, loneliness, and strong feelings of guilt. When asked to address a significant character from their lives through a letter, speak to an empty chair, or use role-playing, the group participants often expressed feelings of guilt. They felt consumed by feelings of guilt toward close friends, partners, and especially to parents, or parents toward their children.

At times, psychodramatic role-playing enabled participants to forgive themselves, at least temporarily:

Raphael begins reading a letter addressed to his son, in which he asks for forgiveness for his illness, and everything his son had to go through as a result over all those years. He says that he hurt his son, that he misses him. As he read, Raphael bursts into tears.

I ask Raphael to reverse roles, and he responds to himself in the place of his son: “I understand your situation, I miss and love you too, Dad.”

The participants saw the opportunity to express distress within the accepting and supportive space of the group as beneficial to them. This space was perceived as better than the outside world. Ella expressed this after telling about the loneliness she was experiencing:

Ella: “Here there’s this togetherness, but in the real world you’re alone. Each person on his own.”

I ask: “What’s ‘the real world’?”

Ella: “Outside.”

I: “What about in this room?”

Ella: “Here it’s better, here you’re not alone.”

I: “And what happens here, can it affect the outside, at least a little?”

Ella: “I hope so.”

### The Doubling Technique in the Therapeutic Process

Alongside the many manifestations of participants’ distress, one could also see that psychodrama can make a group member feel visible, to inspire hope and serve as a space for self-expression, interpersonal encounter and sharing. One of the most powerful therapeutic means in this context was the *double*. The double in psychodrama is meant to act as an additional “I”, which allows a protagonist (the client whose psychodrama is enacted on stage) to express those things that are most difficult to express, to share thoughts and feelings that may be difficult to articulate into words, and introduce repressed conflicts while providing a sense of safety and support. The double echoes the thoughts and feelings of the protagonist, while ensuring a sense of being seen and heard, expressing empathy, closeness, and understanding of the feelings and perspective of the protagonist.

In this group of inpatients, it was almost always the group therapist who acted as the double, as group members rarely doubled for one another. This may be a reflection of the open nature of the group; the constantly changing group membership did not allow for long-term experience with the doubling technique and the confidence to undertake the role of a double for other group members. At times, however, the participants did ask that others would double for them:

David explains to the others what a double is, and I suggest that he demonstrate to the others. He prefers not to, and asks me to double for him.

I speak as his double: “I know exactly what a double is, but I prefer that others double for me. I am little too embarrassed to double for someone else, and I’m afraid of taking that responsibility. What if he won’t agree or won’t like the double I’ve made for him? What if I embarrass him? I don’t want to embarrass anyone or stand out too much.” David smiles and thanks me.

Here, the double not only demonstrated the technique of doubling to the group, but also offered David an interpretation of his own behavior, to clarify and illuminate his thoughts and worries, and help David be better understood by his fellow group-members and himself. Even in this double demonstration, David was offered a message of empathy, an assurance that the therapist heard and understood him. It seemed that this gesture of empathy stayed with David even later in the session:

At the end of the session, the participants walk around the room and wish each other something nice for the day. There is a lot of warmth; David hugs everyone excitedly, then thanks me.

The doubles were used for many purposes throughout the year. At times, the goal was to increase a sense of visibility in participants who felt transparent, invisible:

We return to Clara who unexpectedly begins to describe to the group how sometimes she follows people around the ward, and is often told to stop following them. I double for her: “I feel lost here and no one sees me.”

At times, besides restoring a sense of visibility for the participants, I used the double to give voice to anyone who feels hurt and frustrated yet may not know how to express it in the group:

Mayer speaks angrily in a seemingly disproportionate and extreme manner about the cafeteria that closed today without prior notice, and that this isn’t the first time. He cannot calm down from this.

I double him: “I am angry. They can’t do this, close without warning, without giving me a chance to prepare! And this isn’t the first time. It’s like they just don’t care about us, they completely disregard me. It’s infuriating and frustrating”

Mayer thanks me warmly. It seems he is moved by the double, and his anger subsides.

There were times when the double was used to clarify chaotic situations in the group, relieve stressful or threatening situations, or reduce anxiety for some of the participants. In one session, one of the participants, Eddie, was apparently in a psychotic state when he began to express his feelings toward a woman in the group, in a bizarre, inappropriate, uncontrollable and even threatening manner:

Eddie sits in the empty chair and begins to talk about Naomi (a new participant in the group). He is incoherent, tries to describe how much he loves her, and how he is happy about their connection. “I have patience,” he adds. There is a sense of awkwardness and discomfort in the group; Igor suggests moving on to someone else.

I double for Eddie - “Naomi, I am very glad I’ve met you, it makes me happy that we’ve gotten to know each other, and I may also have some feelings for you.” I ask Naomi if she would like to respond or to say something; she refuses, so I move on to other participants.

Eddie’s remarks were seen as bizarre, uncontrollable, and threatening. The double was used to humanize his experience and to express his feelings in a more coherent and relatable way. This seems to have restored a sense of control and order in the group, alleviate pressure from Naomi who was the object of Eddie’s address, while at the same time allowing Eddie to cool down, and moving on without ignoring or offending him.

Other times, the double seem to have brought group members together and allowed them to express closeness, offering means of expression of empathy between the participants whenever it seemed they were trying to express empathy. Such an opportunity arose in one session right after one participant, Laura, revealed that she had been raped:

Michael says he is usually against the death penalty, but he thinks it is an appropriate punishment for anyone who stabs and rapes someone.

I double for Michael, speaking for him: “I’m telling you this because that’s what I think, but also because I’m trying to tell you, Laura, how much your story touched me. I feel your pain, and I’m here with you.”

Michael responds, “Exactly!”

Michael’s response to Laura’s story took the form of an opinion about the punishment fitting for rapists. Michael’s double enabled him to express himself in a different way – more personal and emotional, and express empathy toward Laura. His response, “Exactly!”, demonstrated that he indeed felt empathy and compassion toward Laura that he was trying to express.

### The Role of Group Sharing

In addition to the use of doubling, the psychodramatic sharing phase, as well as acts of interpersonal sharing throughout the sessions, enabled an experience of universality and mutual support in the group. The classic sharing phase in psychodrama is the phase in which group members share their personal life experiences as they relate to the work of the protagonist. In practice, the psychodrama activity in the group did not always focus on one protagonist, and there was not always a clear separation between the main activity and the sharing phase. Participants in the group could share their feelings, their troubles, and whatever else they were undergoing. This space often evoked an experience of universality; a discovery that the individual is not alone in his experience and in his distress:

The group members share how they are doing this morning. Yehuda shares that he is confused about his place in the world. When he doesn’t observe the commandments of Judaism he feels emptiness, yet when he tries to observe the rituals he becomes unbalanced and triggers manic episodes. Another participant, Abraham tells him that when a person brings oneself closer to religion, at first God gives a push forward, but afterward one is left [to struggle] with it alone. I ask Abraham if this is something he has personally experienced; he says yes, and adds: “We are souls attached together, souls that speak.”

In another example, a group sharing, occurred after a psychodynamic vignette, provided an experience of universality when one of the participants shared her feelings of guilt with the group:

Alice describes the great difficulty she causes her mother because of her illness. [She tells about] the guilt she feels toward her mother. She becomes emotional and cries.

We discuss feelings of guilt surrounding illness, as Alice has expressed. How it is an experience that other participants share as well. Elena and Rachel say they also feel guilt toward their families.

…At the end of the session Alice hugs Elena. She thanks her and says she feels much better now.

When a particularly painful sentiment was offered during the group sharing, it was frequently possible to see how the participants tried to offer support, empower, and encourage one another, say a good word, and to offer solutions, perspectives, or suggest an alternative approach:

Jacob (another new participant) begins to tell about himself as well… He tells about his three daughters and several grandchildren, how none of them know that he is hospitalized here. That he especially doesn’t want his sons-in-law to find out. One of them is a doctor in a university, another is an engineer.

Diane asks to tell Jacob that he should tell them all that he is hospitalized, that he shouldn’t feel ashamed. That she was once in a closed psychiatric ward, and “the best attorney in the country” was there too. Jacob smiles and thanks Diane.

Anna tells the group about her suicidal thoughts: “I’ve been here for 2 months already and nothing is better, I want to die every day, but I promised that as long as I’m here I will not do anything…People always tell me I look better, but I don’t feel better.” Sharon says that she sees that Anna is suffering and is depressed, and she wishes for her to feel better. She says Anna is lovely and deserves to feel well.

Isaac also speaks of depression of despair over destroying himself and losing everything, of great fatigue. He says he just wants to hide from reality and sleep. David talks about faith and tries to encourage Isaac.

Here we can see how the group sharing has allowed to create a space of mutual support: Sharon tries supporting Anna and David encourages Isaac. Later Anna and Isaac receive additional gestures of encouragement and support from the group. It was not always clear whether the distress heard was the personal distress of Anna or Isaac, and to what extent these empowering words belonged to David or Sharon. Sometimes it seemed that all of these voices were the voice of the group itself; that each participant exhibits distress and need, at times despair, alongside great strength, optimism and the desire to help. Sharing within the group enabled an important space for expression of these voices and for dialog between them.

Sometimes, when group members expressed distress and painful feelings, the group sharing acquired a certain ceremonial status that enabled a shared experience of dealing with the distress. This was demonstrated later in the same session described above in which Anna and Isaac shared their suffering with the group. The following sharing segment took on a different character than usual:

We place a circle of hoops in the room, and each hoop represents a feeling that arose during the encounter: sadness, confusion, optimism, depression, comradeship, joy, shame, fatigue… Each participant chooses a hoop and tells the group what he would like to do with it.

Boris chooses the hoop labeled “despair,” to tell Anna not to lose hope. Isaac chooses (David’s) optimism. Yehuda chooses faith and optimism. He addresses his words to Anna and Isaac, and talks about God, who, even if it is difficult to understand, always has our interests in mind.

Finally, I take the comradeship hoop, and say that I’ve felt a lot of that comradeship within the last hour.

Here, the hoops allowed the participants to give away or take from each other some of their strengths as well as their distress. This was a powerful manifestation of the concept of sharing – dividing the stresses and emotions among the group. The concept was best expressed by Laura at the end of a difficult session during which she shared the story of her rape with the group:

Sharing: I ask the participants to mention one thing they will take with them when they leave the room. Most of the participants wish for Laura to feel better, healthy and strong.

Laura herself says: “I take some of the pain of each one in the group.”

I ask, as Laura’s double, “Do I also take with me some of the group’s love?”

Laura: “I’ll try.”

I, as Laura’s double: “Do I see the group’s love?”

Laura: “Yes”

Laura’s statement that she takes “some of the pain of each one in the group,” together with the double that invites Laura to also take “some of the group’s love,” concisely expresses the concept of sharing - distribution of the heavy burden, as well as the resources and strengths of the participants in the group. This distribution is the essence of the receptacle created by our psychodrama group; a chamber of cooperation, reciprocity and human interaction that may, to some extent, alleviate the distress of psychiatric inpatients.

## Discussion

The findings of this study illustrate a picture of acute distress of psychiatric inpatients in Israel, but also of an experience of relatedness, mutual support, and human encounter in group psychodrama that seem to have enabled participants to express and cope with this distress.

Manifestations of distress described in the paper include expressions of depression, despair, helplessness and weariness, loss of will to live, and self-labeling of the participants. These descriptions reinforce the findings from research of symptoms that arise in people following a medical diagnosis (Moore et al., 1999; Roe and Lachman, 2005), descriptions by Schur (1971) of people coping with SMI being “engulfed by their patient-role,” as well as the findings of self-stigma and learned helplessness, loss of self-belief, despair, and loss of will which characterize the experience of coping with mental illness (Deegan, 2004; Yanos et al., 2011; Orkibi et al., 2014).

Furthermore, people hospitalized in a psychiatric hospital must also cope with feelings of guilt toward friends and relatives, and suffer the loneliness, loss of independence, and the distress associated with psychiatric hospitalization (Morrison et al., 2003; Holloway, 2010). Manifestations of such feelings; of loneliness, isolation and feelings of guilt toward close friends, partners, and especially toward parents or children of the participants, were frequently manifested in the inpatients psychodrama group, as described in the findings section.

Besides the description of the participants’ acute distress manifested in the therapeutic process, this study examines how the psychodrama group enabled participants to cope with their distress by creating a space for self-expression and a human encounter, mutual support, and sharing. The study illustrates the role of the psychodramatic “double”: its capacity to echo thoughts and emotions of participants, offer commentary and interpretation, and help participants feel they are better understood. The double was employed as a way to give a voice to group members who struggled to express themselves, and to increase clarity in chaotic situations, reduce anxiety, and enable expressions of identification, empathy, and closeness among group members. This reflects the concepts of Moreno and his successors of the double as an “additional I,” which allows the protagonist a sense of visibility and facilitate expression of thoughts and feelings; the doubling technique is aimed at helping the participant to confront repressed conflicts, and seeks to provide a supportive environment and sense of safety in the psychodramatic space. This method relies on the empathy and sense of closeness and understanding between participants (Holmes and Karp, 1991; Blatner, 2000; Fox, 2008).

Next, the significant role of sharing in psychodrama was examined in this study; this refers not only to the sharing phase after the psychodramatic action, but also to other moments of sharing at the interpersonal level that occur during the group session. The study illustrates how group sharing may facilitate visibility, mutual support, and interpersonal encounter. In the inpatients psychodrama group, participants could share themselves with the group, their feelings and thoughts, and sense the attentiveness of the other participants, who occasionally offered their responses as well. Sharing seemed to enable Group members a sense of universality (Yalom, 1983), or “mirror reaction” as termed by Foulkes (Fehr, 2003), in which participants discover that they are not alone in their distress; that their fellow group members cope with similar distress and they share it with the group. The sharing also enabled dialog between group members, about their experiences, values, thoughts, or emotions. For inpatients this may be one of the few places where they can encourage, compliment, or support one another, express their identification, offer alternative perspectives, or just listen empathetically. Here in this space, many of Yalom’s therapeutic factors manifested themselves – such as instillation of hope, universality, group cohesiveness and altruism (Yalom, 1983, 1995).

Moreno described the sharing phase as a phase where “strangers” in the group can reveal their emotions and cease to be strangers, can express their love for the protagonist, and allow for their own self-expression (Moreno, 1946, in Fox, 2008). This study demonstrates how group participants used sharing to distribute the burden among the group members along with the resources to cope with it. This is the essence of what Moreno described as the fabric of life and human encounter which comprises the psychodrama group (Moreno, 1939, 1946; Blatner, 2000, in Moreno, 1953; Fox, 2008), allowing its members to experience empathy, relatedness and support, which offers at least partial relief of the distress and loneliness of psychiatric inpatients.

## Limitations of the Study and Future Directions

The above findings may contribute to our understanding of the potential of psychodrama in treating psychiatric inpatients, but this study is not without limitations. First, the descriptive nature of this study is not intended to provide a precise measurement of the effects of psychodrama therapy or the level of distress in participants, nor does it purport to definitively demonstrate a direct causal relationship between these two factors. Instead, like other cases of clinical case reports, this study relies on anecdotal data in which clinical judgment and interpretation play a major role. Alternative explanations are available to account for the processes and changes described other than those provided here. In order to achieve a more complete picture there is clearly room for further research, including quantitative studies that can produce reproducible and generalizable results regarding the effects of therapeutic means such as the doubling technique and group sharing phase, on the level of distress of psychiatric inpatients.

An additional methodological issue concerns the danger of blurring boundaries between researcher and practitioner, and between research and clinical work. Nevertheless, case study research offers significant advantages – its relevance as applied research, as well as the deep familiarity of the researcher with the participants. In this case this familiarity enables an intimate and unique encounter with psychiatric inpatients’ acute distress and with the therapeutic processes that arise in the psychodramatic space.

## Conclusion

The current study contributes to our understanding of the potential of psychodrama group therapy in dealing with the distress and isolation of acute psychiatric inpatients. Current literature describes how the unique features of psychodrama therapy are useful in fostering spontaneity and creativity (Farmer, 1995; Roine, 1997; Blatner, 2000; Schacht, 2007), and in treating particularly difficult populations where traditional psychotherapy is limited (Karp, 1994; Vieira and Risques, 2007; Karatas, 2011; Orkibi et al., 2017). The unique contribution of this study is the intimate encounter that it provides to researchers and practitioners through holistic and naturalistic, in-depth investigation in a real-life setting, with manifestations of the distress accompanying psychiatric hospitalization, the processes that take place within an inpatients therapy group, and with therapeutic dimensions such as psychodramatic sharing, which are rarely dealt with by the existing literature.

## Data Availability Statement

The datasets for this manuscript are not publicly available due to reasons of confidentiality and participants’ privacy. Requests to access the datasets should be directed to YR, yiftach.ron@smkb.ac.il.

## Ethics Statement

An ethics approval for this study was not required as per the authors’ institutions guidelines and national regulations. Written informed consent was obtained from all participants.

## Author Contributions

YR data collection and analysis, and writing of the manuscript.

## Conflict of Interest Statement

The author declares that the research was conducted in the absence of any commercial or financial relationships that could be construed as a potential conflict of interest.

## Footnotes

1 Although they are often used interchangeably, the term “open-ended” can more precisely refer to the timeframe of the group, while “open” refers to the membership of the group (Turner, 2011).

## References

[B1] Belil F. E. (2010). The effects of psychodrama on depression and mental state among women with chronic mental disorder. *Eur. Psychiatry* 25:1049 10.1016/S0924-9338(10)71039-7

[B2] Berg B. L. (2004). *Qualitative Research Methods for the Social Sciences.* Boston, MA: Pearson.

[B3] Blatner A. (2000). *Foundations of Psychodrama: History, Theory and Practice*, 4th Edn New York, NY: Springer.

[B4] Corrigan P. W. (2004). How stigma interferes with mental health care. *Am. Psychol.* 59 614–625. 10.1037/0003-066X.59.7.614 15491256

[B5] Corrigan P. W. Rao D. (2012). On the self-stigma of mental illness: stages, disclosure, and strategies for change. *Can. J. Psychiatry* 57 464–469. 10.1177/070674371205700804 22854028 PMC3610943

[B6] Crowe S. Cresswell K. Robertson A. Huby G. Avery A. Sheikh A. (2011). The case study approach. *BMC Med. Res. Methodol.* 11:100. 10.1186/1471-2288-11-100 21707982 PMC3141799

[B7] Deegan P. E. (2004). “Rethinking rehabilitation: freedom,” in *Proceedings of the the 20th World Congress of Rehabilitation International: Rethinking Rehabilitation*, Tartu.

[B8] Ebenstein H. (1998). Single-Session groups: issues for social workers. *Soc. Work Groups* 21 49–60. 10.1300/J009v21n01_05

[B9] Fagin L. (2010). “Is it possible to make acute wards into therapeutic communities,” in *Psychological Groupwork with Acute Psychiatric Inpatients*, eds Radcliffe J. Hajek K. Carson J. Manor O. (London: Whiting & Birch),21–36.

[B10] Farmer E. (1995). *Psychodrama and Systemic Therapy.* London: H. Karnac.

[B11] Fehr S. S. (2003). *Introduction to Group Therapy: A Practical Guide.* New York, NY: Haworth Press.

[B12] Fox J. (2008). *The Essential Moreno: Writings on Psychodrama, Group Method, and Spontaneity, by J. L. Moreno.* New Paltz, NY: Tusitala Publishing.

[B13] Frame L. Morrison A. P. (2001). Causes of posttraumatic stress disorder in psychosis. *Arch. Gen. Psychiatry* 58 305–306. 10.1001/archpsyc.58.3.305 11231839

[B14] Gatta M. Dal Zotto L. Del Col L. Spoto A. Testa C. P. Ceranto G. (2010). Analytical psychodrama with adolescents suffering from psycho-behavioral disorder: short-term effects on psychiatric symptoms. *Arts Psychother.* 37 240–247. 10.1016/j.aip.2010.04.010

[B15] Harrison H. Birks M. Franklin R. Mills J. (2017). Case study research: foundations and methodological orientations. *Forum Qual. Soc. Res.* 18 1–17. 10.17169/fqs-18.1.2655 8053253

[B16] Hollander E. M. Craig M. (2013). Working with sexual offenders via psychodrama (in US, Colorado). *Sex. Offend. Treat.* 8 Available at: http://www.sexual-offender-treatment.org/124.html

[B17] Holloway F. (2010). “Acute wards: contexts, pressures and satisfactions,” in *Psychological Groupwork with Acute Psychiatric Inpatients*, eds Radcliffe J. Hajek K. Carson J. Manor O. (London: Whiting & Birch), 51–70. 10.1080/01609513.2010.508623

[B18] Holmes P. Karp M. (1991). “Inspiration and technique,” in *Psychodrama: Inspiration and Technique*, eds Holmes P. Karp M. (London: Routledge), 1–6.

[B19] Kanas N. (1996). *Group Therapy for Schizophrenic Patients.* Washington, DC: American Psychiatric Press.

[B20] Karatas Z. (2011). Investigating the effects of group practice performed using psychodrama techniques on adolescents’ conflict resolution skills. *Educ. Sci.* 11 609–614.

[B21] Karp M. (1994). “The river of freedom,” in *Psychodrama Since Moreno: Innovations in Theory and Practice*, eds Holmes P. Karp M. Watson M. (London: Routledge), 39–60.

[B22] Kazdin A. E. (2011). *Single-case Research Designs: Methods for Clinical and APPLIED settings*, 2nd Edn New York, NY: Oxford University Press.

[B23] Kellermann P. F. (1998). Diagnosis in psychodrama? *Psychodrama* 5 25–32.

[B24] Lysaker P. H. Lysaker J. T. (2004). “Dialogical Transformation in the psychotherapy of Schizophrenia,” in *The Dialogical Self in Psychotherapy*, eds Hermans Dimaggio (New York, NY: Brunner-Routledge), 205–219. 10.4324/9780203314616_chapter_13

[B25] Mankiewicz P. D. Gresswell M. Turner C. (2013). Happiness in severe mental illness: exploring subjective wellbeing of individuals with psychosis and encouraging socially inclusive multidisciplinary practice. *Ment. Health Soc. Inclus.* 17 27–34. 10.1108/20428301311305287

[B26] Manor O. (2010). “The single session format: common features groupwork in acute psychiatric wards,” in *Psychological Groupwork with Acute Psychiatric Inpatients*, eds Radcliffe J. Hajek K. Carson J. Manor O. (London: Whiting & Birch), 132–154. 10.1080/01609513.2010.508623

[B27] McVea C. S. Gow V. Lowe R. (2011). Corrective interpersonal experience in psychodrama group therapy: a comprehensive process analysis of significant therapeutic events. *Psychother. Res.* 21 416–429. 10.1080/10503307.2011.577823 21623548

[B28] Miller R. Mason S. E. (2012). Open-ended and open-door treatment groups for young people with mental illness. *Soc. Work Groups* 35 50–67. 10.1080/01609513.2011.587099 22427713 PMC3305594

[B29] Moore O. Cassidy E. Carr A. O’Callaghan E. (1999). Unawareness of illness and its relationship with depression and self-deception in schizophrenia. *Eur. Psychiatry* 14 264–269. 10.1016/S0924-9338(99)00172-8 10572356

[B30] Moreno J. L. (1939). “Psychodramatic treatment of psychoses,” in *The Essential Moreno: Writings on Psychodrama, Group Method, and Spontaneity by J. L. Moreno*, ed. Fox J. (New Paltz, NY: Tusitala Publishing), 71–84.

[B31] Moreno J. L. (1946). “Psychodrama and sociodrama,” in *The Essential Moreno: Writings on Psychodrama, Group Method, and Spontaneity by J. L. Moreno*, ed. Fox J. (New Paltz, NY: Tusitala Publishing), 13–19.

[B32] Moreno J. L. (1953). *Who Shall Survive? Foundations of Sociometry, Group Psychotherapy, and Sociodrama*, 2nd Edn Beacon, NY: Beacon House.

[B33] Morrison A. P. Frame L. Larkin W. (2003). Relationships between Trauma and Psychosis: a review and integration. *Br. J. Clin. Psychol.* 42 331–353. 10.1348/014466503322528892 14633411

[B34] Mueser K. T. Lu W. Rosenberg S. D. Wolfe R. (2010). The trauma of psychosis: posttraumatic stress disorder and recent oncet psychosis. *Schizophr. Res.* 116 217–227. 10.1016/j.schres.2009.10.025 19939633

[B35] Mueser K. T. Rosenberg S. D. Goodman L. A. Trumbetta S. L. (2002). Trauma, PTSD, and the course of severe mental illness: an interactive model. *Schizophr. Res.* 53 123–143. 10.1016/S0920-9964(01)00173-6 11728845

[B36] Orkibi H. Azoulay B. Snir S. Regev D. (2017). In-session behaviors and adolescents’ self-concept and loneliness: a psychodrama process-outcome study. *Clin. Psychol. Psychother.* 24 1455–1463. 10.1002/cpp.2103 28653318

[B37] Orkibi H. Bar N. Elkayam I. (2014). The effect of drama-based group therapy on aspects of mental illness stigma. *Arts Psychother.* 41 458–466. 10.1016/j.aip.2014.08.006

[B38] Roe D. Lachman M. (2005). The subjective experience of people with severe mental illness: a potentially crucial piece of the puzzle. *Israel J. Psychiatry Relat. Sci.* 42 223–230. 16618053

[B39] Roine E. (1997). *Psychodrama: Group Psychotherapy as Experimental Theatre – Playing the Leading Role in Your Own Life.* London: Jessica Kingsley Publishers.

[B40] Ron Y. (2018). The qualities of psychodramatic group work with inpatients in psychiatric hospitals: a literature Review. *Bein Hamilim* 14 (in Hebrew) Available at: https://www.smkb.ac.il/arts-therapy/beyn-hamilim14/artical/ben-hamilim14-ron

[B41] Roth W. M. (2005). *Doing Qualitative Research: Praxis of Method.* Rotterdam: Sense.

[B42] Schacht M. (2007). “Spontaneity-Creativity: the psychodramatic concept of change,” in *Psychodrama: Advances in Theory and Practice*, eds Baim C. Burmeister J. Maciel M. (New York NY: Taylor & Francis), 21–40.

[B43] Schopler J. H. Galinsky M. J. (1984/2006). Meeting practice needs: conceptualizing the open-ended group. *Soc. Work Groups* 28 49–68. 10.1300/J009v28n03_05

[B44] Schur E. M. (1971). *Labeling Deviant Behavior: Its Sociological Implications.* New York, NY: Harper & Row.

[B45] Shaw K. McFarlane A. Bookless C. Air T. (2002). The aetiology of postpsychotic posttraumatic stress disorder following a psychotic episode. *J. Trauma Stress* 15 39–47. 10.1023/A:1014331211311 11936721

[B46] Shulman L. (2006). *The Skills of Helping Individuals, Families, Groups, and Communities*, 5th Edn Belmont, CA: Thomson Brooks/Cole.

[B47] Stake R. E. (1995). *The Art of Case Study Research.* Thousand Oaks, CA: Sage.

[B48] Strauss A. Corbin J. (1998). *Basics of Qualitative Research: Techniques and Procedures for Developing Grounded Theory.* Thousand Oaks, CA: Sage.

[B49] Turner H. (2011). Concepts for effective facilitation of open groups. *Soc. Work Groups* 34 246–256. 10.1080/01609513.2011.558822

[B50] Vieira F. Risques M. (2007). “Psychodrama and psychopathology: purposefully adapting the method to address different pathologies,” in *Psychodrama: Advances in Theory and Practice*, eds Baim C. Burmeister J. Maciel M. (New York NY: Taylor & Francis), 247–260.

[B51] Whitaker D. S. (2001). *Using Groups to Help People*, 2nd Edn New York, NY: Brunner-Routledge.

[B52] Yalom I. D. (1983). *Inpatient Group Psychotherapy.* New York, NY: Basic Books.

[B53] Yalom I. D. (1995). *The Theory and Practice of Group Psychotherapy.* New York, NY: Basic Books.

[B54] Yanos P. T. Roe D. Lysaker P. H. (2011). Narrative enhancement and cognitive therapy: a new group-based treatment for internalized stigma among persons with severe mental illness. *J. Group Psychother.* 61 577–595. 10.1521/ijgp.2011.61.4.576 21985260 PMC3191919

[B55] Yin R. K. (2009). *Case Study Research: Design and Methods*, 4th Edn Thousand Oaks, CA: Sage.

